# Elucidating Key Components and Mechanisms Underlying the Synergistic Anti-Type 2 Diabetes Effect of *Morus alba* L. and *Siraitia grosvenorii* Combination: An Integrated In Vitro Enzymology, Untargeted Metabolomics, and Network Pharmacology Approach

**DOI:** 10.3390/antiox14091065

**Published:** 2025-08-29

**Authors:** Fang He, Shenglan Su, Ruihan Song, Yan Li, Luyan Zou, Zongjun Li, Yu Xiao, Aixiang Hou, Ke Li, Yuanliang Wang

**Affiliations:** 1 College of Food Science and Technology, Hunan Agricultural University, Changsha 410128, China; fanghe_0615@163.com (F.H.); sushenglan@stu.hunau.edu.cn (S.S.); 13407319765@stu.hunau.edu.cn (R.S.); liyanlucky@stu.hunau.edu.cn (Y.L.); zly@stu.hunau.edu.cn (L.Z.); lizongjun@hunau.edu.cn (Z.L.); xiaoyu@hunau.edu.cn (Y.X.); aixianghou@hunau.edu.cn (A.H.);; 2 Hunan Province Key Laboratory of Food Science and Biotechnology, Changsha 410128, China

**Keywords:** mulberry leaf, *Siraitia grosvenorii*, type 2 diabetes, untargeted metabolomics, network pharmacology, functional food

## Abstract

Although mulberry leaf (*Morus alba* L., ML) and *Siraitia grosvenorii* (SG) individually demonstrate anti-diabetic properties, their combined efficacy against type 2 diabetes mellitus (T2DM) remains unexplored. This study systematically explored the multi-target mechanisms and synergistic potential of the MLSG combination (MLSG) for T2DM intervention. We evaluated the in vitro inhibitory activities of MLSG, ML, and SG on α-amylase and α-glucosidase, alongside antioxidant capacity assessments through DPPH/ABTS radical scavenging, reducing power, and FRAP assays. Bioactive metabolites were identified using non-targeted metabolomics, while core targets and pathways were predicted using network pharmacology and validated through molecular docking. The results reveal MLSG’s significantly enhanced inhibition of α-amylase (IC_50_ = 14.06 mg/mL) and α-glucosidase (IC_50_ = 0.02 mg/mL) compared to individual extracts, exhibiting 1.3–15.5-fold higher potency with synergistic effects (combination index < 1). MLSG also showed improved antioxidant capacity, outperforming SG in DPPH/ABTS^+^ scavenging and reducing power (*p* < 0.05), and surpassing ML in ABTS^+^ scavenging, reducing power, and FRAP values (*p* < 0.05). Metabolomics identified 26 MLSG-derived metabolites with anti-T2DM potential, and network analysis pinpointed 26 active components primarily targeting STAT3, AKT1, PIK3CA, EGFR, and MAPK1 to regulate T2DM pathways. Molecular docking confirmed strong binding affinities between these components and core targets. Collectively, MLSG exerts potent synergistic anti-T2DM effects through dual-enzyme inhibition, elevated antioxidant activity, and multi-target pathway regulation, providing a solid foundation for developing MLSG as functional food ingredients.

## 1. Introduction

Type 2 diabetes mellitus (T2DM) represents a rapidly expanding global metabolic pandemic, currently affecting over 424 million individuals, with projections soaring to 627 million by 2045 [1]. Consequent cardiovascular and renal complications have tripled healthcare expenditures [1]. While first-line therapeutics, such as metformin, provide glycemic control, 30% of patients endure gastrointestinal adverse effects, and insulin sensitizers, such as rosiglitazone, face restricted use due to cardiovascular risks [2,3]. Consequently, developing safe interventions that concurrently regulate glucolipid metabolism and preserve pancreatic islet function remains an urgent unmet medical need.

Natural products, with multi-target pharmacology and favorable safety, have re-emerged as promising candidates [4]. This potential is exemplified by synergistic botanical combinations: the Scutellaria–Coptis pair restores glycemic control through multi-mechanistic actions in diabetic models [5], while bitter melon–mulberry composites alleviate obesity-associated metabolic dysregulation via gut-barrier enhancement [6]. Over 40% of diabetes patients worldwide use phytomedicines [7]. Mulberry leaf (*Morus alba* L.) and monk fruit (*Siraitia grosvenorii*) epitomize China’s “medicinal food” tradition, demonstrating significant anti-diabetic potential. Mulberry leaf multi-components ameliorated hepatic glucolipid metabolism disorders and improved insulin resistance in T2DM rats by activating the PI-3K/Akt signaling pathway [8]. Mulberry’s 1-deoxynojirimycin (DNJ) inhibits α-glucosidase (IC_50_ = 0.7 μg/mL), and flavonoids provide potent anti-inflammatory and antioxidant properties [9,10]. Similarly, mogrosides extracted from monk fruit (MGE) exhibit potential as a novel anti-glycation agent for mitigating diabetic complications. Mogroside-rich extract (MGE) significantly reduces fasting blood glucose, glycated serum protein, and serum insulin levels in a dose-dependent manner, potentially through attenuating insulin resistance and activating hepatic AMPK signaling in diabetic models [11]. In HFD mice, MGE’s hypoglycemic effect also result from gut microbiota modulation, improving glucose tolerance [12]. However, the current research exhibits significant limitations: (1) the anti-T2DM and antioxidant efficacy of their combinatorial extracts application has never been quantitatively assessed; and (2) the mechanistic basis underlying their combined anti-T2DM effects remains unknown. This critical knowledge gap impedes the translation of traditional empirical knowledge into evidence-based applications.

Modern systems biology approaches now enable the deconvolution of such complex synergies. Untargeted metabolomics via UHPLC-QTOF-MS can profile > 5000 metabolites with <5 ppm mass accuracy [13]. When integrated with network pharmacology, which constructs multi-dimensional “compound–target–pathway” interactions via databases like OMIM and GeneCards, researchers can identify core therapeutic targets and pathway clusters [14]. Molecular docking further validates binding affinities through rigorous free energy calculations (ΔG ≤ −7.0 kcal/mol), with algorithms like AutoDock Vina achieving <0.5 kcal/mol error margins [15]. This integrated strategy overcomes critical limitations of reductionist approaches that overlook synergistic effects and fail to verify computational predictions.

**Figure 1:**
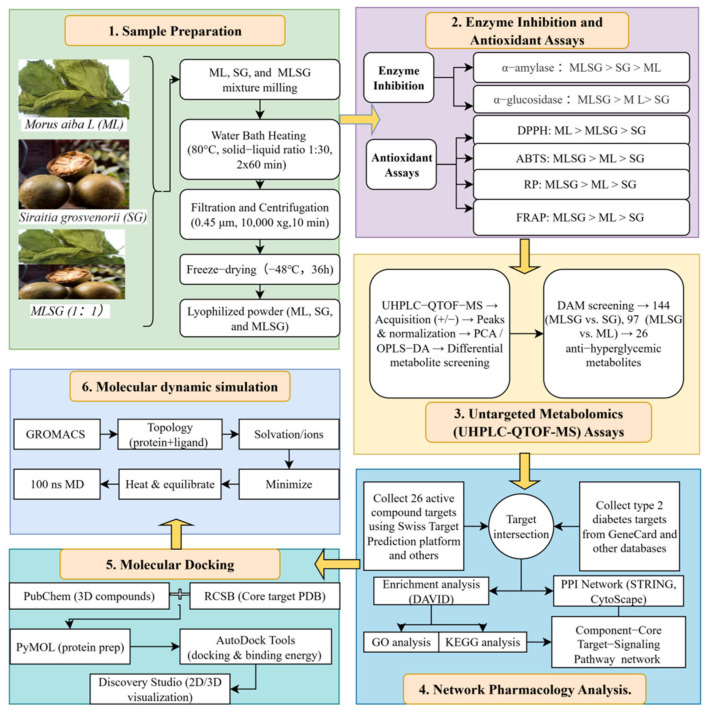
Research methodology workflow. This figure presents the overall workflow of the research, highlighting key stages from sample preparation to network pharmacology analysis. The process integrates enzyme inhibition, antioxidant assays, untargeted metabolomics, molecular docking, and dynamics simulations, leading to the identification of bioactive metabolites with potential effects on improving type 2 diabetes.

Here, we integrate enzyme inhibition, antioxidant assays, UHPLC-QTOF-MS metabolomics, network pharmacology, and molecular docking to ① compare mulberry leaf–*Siraitia grosvenorii* lyophilized powder (MLSG) vs. individual components for enzyme inhibition and antioxidant activity; ② screen and identify clusters of bioactive compounds with anti-T2DM potential; ③ elucidate the molecular basis underlying MLSG’s multi-target synergistic regulation of T2DM (Figure 1). Finally, a mechanistic foundation and design framework will be established to guide the development of evidence-based functional foods or natural therapeutics targeting the pathogenesis of T2DM.

## 2. Materials and Methods

### 2.1. Materials and Chemicals

α-glucosidase (100 U/mg, Cat. No. G0660), p-nitrophenyl-α-glucopyranoside (p-NPG, Cat. No. 487506), Folin–Ciocalteu’s reagent (Cat. No. F9252), diammonium 2,2-azino-bis (3-ethylbenzothiazoline-6-sulfonate) (ABTS, Cat. No. A1888), 2,2-diphenyl-1-picrylhydrazyl (DPPH, Cat. No. 257621), and 2,4,6-tris (2-pyridyl)-S-triazine (TPTZ, Cat. No. T1253) were supplied by Sigma-Aldrich Co. (St Louis, MO, USA). α-amylase (4000 U/g, Cat. No. S31302-50ku) was purchased from Shanghai Yuanye Bio-Technology Co., Ltd. (Shanghai, China). Acarbose (Cat. No. A129816) and soluble starch (Cat. No. S104454) were purchased from Shanghai Aladdin Biochemical Technology Co., Ltd. (Shanghai, China). The other chemicals and reagents were of analytical grade and were purchased from Sinopharm Chemical Reagent Co., Ltd. (Shanghai, China).

Mulberry leaf and *Siraitia grosvenorii* materials were provided by Hunan Runtian Pharmaceutical Company Limited. (Changsha, Hunan, China), with batch numbers 202401-03 and 202403-12, respectively. These raw materials were crushed and subjected to water bath-heated reflux extraction (solid-to liquid-ratio of 1:30, *w*/*v*) at 80 °C for two cycles of 60 min each. The extracts of ML, SG, and a 1:1 mixture (MLSG) were filtered through a 0.45 μm nylon membrane and centrifuged at 10,000× *g* for 10 min. The supernatant was concentrated using a rotary evaporator, followed by lyophilization under vacuum for 48 h to produce lyophilized powders, which were stored at −20 °C.

### 2.2. Measurement of α-Amylase and α-Glucosidase Inhibitory Activity

The determination of α-glucosidase and α-amylase inhibitory activities was optimized using a colorimetric method as described in reference [16]. Briefly, mulberry leaf extract (ML), *Siraitia grosvenorii* extract (SG), and their complex (MLSG) were pre-incubated with α-glucosidase (1 U/mL) or α-amylase (0.4 U/mL) at 37 °C for 10 min. The reactions were initiated by adding 2.5 mM p-nitrophenyl-α-D-glucopyranoside (PNPG) or 0.5% soluble starch (pH 6.8 phosphate buffer). After terminating the enzymatic reaction, absorbance was measured at 405 nm (PNP release) and 540 nm (DNS assay). The inhibition rate was calculated using the following formula: Inhibition Rate = [(Ablank − (Asample − Acontrol))/Ablank] × 100. Acarbose was used as the positive control, and IC50 values were calculated using GraphPadPrism 9.0, with all experiments repeated independently three times.

### 2.3. Combination Index (CI) Calculation

After evaluating the compatibility of multiple drugs, their interactions can be categorized into synergistic, additive, and antagonistic effects. Based on the principles of drug interaction, the combination index (CI) serves as an indicator for the interaction of multiple drugs, where CI < 1, =1, and >1 represent synergistic, additive, and antagonistic effects, respectively [17].

$$CI=\frac{(D{)}_{1}}{(Dx{)}_{1}}+\frac{(D{)}_{2}}{(Dx{)}_{2}}$$

In the formula, (D)_1_ and (D)_2_ represent the concentrations of drug 1 and drug 2 that achieve an inhibition rate of 50% when used in combination, while (Dx_)1_ and (Dx)_2_ represent the concentrations of drug 1 and drug 2 that achieve an inhibition rate of 50% when used individually.

### 2.4. Evaluation of Antioxidant Activities

The antioxidant activity of the samples was evaluated through several assays with different mechanisms. The reducing power (RP), DPPH radical scavenging activity (DPPH), and ABTS cation radical scavenging activity (ABTS) were measured following [18]. Calibration curves were plotted using different concentrations of vitamin C. Results were expressed as milligrams of vitamin C equivalents per gram of dry weight of water extract powder (mg VCE/g d.w.). A ferric reducing antioxidant power (FRAP) assay based on Fe^3+^-TPTZ reduction was assessed using the previously published method, with results expressed as milligrams of FeSO4 equivalents per gram of dry weight of water extract powder (mg FeSO4 equivalent/g d.w.) [19].

### 2.5. Metabolomics Analysis

Samples were homogenized in 600 μLH methanol containing 2-chloro-L-phenylalanine (4 ppm, internal standard) using steel beads (55 Hz, 60 s), followed by vortexing (30 s), ultrasonication (15 min, 25 °C), and centrifugation (12,000 rpm, 10 min, 4 °C). The supernatant was filtered through a 0.22 μm membrane for LC-MS analysis. LC–MS data acquisition and preliminary processing were performed by Panomix Biomedical Tech Co., Ltd. (Suzhou, China), according to their standard operating procedures. Raw LC–MS data were converted into mzXML format using ProteoWizard MSConvert (v3.0.8789). Metabolites were annotated by matching exact mass (±5 ppm) and MS/MS spectra against HMDB, MassBank, LipidMaps, KEGG, and an in-house database (Panomix Biomedical Technology Company Limited, Suzhou, Jiangsu, China)) with predicted molecular formulae based on adduct ions and fragmentation patterns. Multivariate analysis (PCA, OPLS-DA) was performed using the Metware Cloud, a free online platform for data analysis (https://cloud.metware.cn, accessed on 7 August 2025). Differential metabolites were screened by VIP > 1 (OPLS-DA), fold change (FC) > 2, and *p*-value < 0.05 (Student’s *t*-test).

### 2.6. MLSG Treatment of T2DM Target Acquisition

Canonical SMILES, molecular weights, and molecular formulae of MLSG compounds were retrieved from the PubChem database (https://pubchem.ncbi.nlm.nih.gov, accessed on 10 February 2025), followed by target prediction using the Swiss Target Prediction platform (http://swisstargetprediction.ch, accessed on 15 February 2025; probability score > 0) and the SEA database (http://sea.bkslab.org, accessed on 15 February 2025). T2DM-related targets were curated from the TTD (https://idrblab.org/ttd, accessed on 20 February 2025), OMIM (http://www.omim.org, accessed on 22 February 2025), and GeneCards (https://www.genecards.org, accessed on 25 February 2025) databases, retaining entries with correlation scores above database-specific averages and filtering for human orthologs in UniProt (http://www.uniprot.org, accessed on 1 March 2025). A Venn analysis (https://bioinfogp.cnb.csic.es/tools/venny/index.html, accessed on 5 March 2025) was performed to identify overlap between MLSG-predicted targets and T2DM-associated proteins, thereby defining the core therapeutic targets regulated by MLSG in T2DM.

### 2.7. Construction of Protein–Protein Interaction (PPI) Network

To collect the overlapping drug and disease targets, we imported the potential drug targets and T2DM targets from MLSG into the Venn database (https://bioinfogp.cnb.csic.es/tools/venny/index.html, accessed on 5 March 2025). The overlapping targets were imported into the STRING database (https://cn.string-db.org, accessed on 10 March 2025) to obtain protein interaction network information. The organism screening condition was set to “Homo sapiens”, and the minimum required interaction score was “highest confidence (0.9)”. The protein–protein interaction (PPI) information was entered into Cytoscape (version 3.10.0), the targets with a degree value greater than the median were selected for visualization, and a PPI network was constructed. The CytoNCA plug-ins were then used to obtain key modules and screen key targets.

### 2.8. Construction of “Component–Core Target–Signaling Pathway” Network

The collected active components and potential targets from MLSG were imported into Cytoscape (version 3.10.0) to construct a component–core target–signaling pathway network. The CytoNCA tool was used to calculate the topological parameters, and then the component–target–pathway network was analyzed based on the degree value.

### 2.9. GO Enrichment and KEGG Pathway Analysis

To study the biological function of potential targets in T2DM, the DAVID database (https://david.ncifcrf.gov/, accessed on 13 March 2025) was used to collect GO analysis and KEGG data. GO analysis was used to screen biological processes (BPs), cellular components (CCs), and molecular functions (MFs). KEGG enrichment analysis can find important signaling pathways involved in biological processes. The GO and KEGG data were then uploaded to the bioinformatics platform (http://www.bioinformatics.com.cn/, accessed on 13 March 2025) for visual analysis.

### 2.10. Molecular Docking and ADMET Analysis

The 3D structures of the top 10 MLSG bioactive compounds were obtained from PubChem (https://pubchem.ncbi.nlm.nih.gov, accessed on 3 August 2025), and crystal structures of five core targets—STAT3 (PDB: 6njs), PIK3CA (PDB: 4jps), AKT1 (PDB: 3o96), EGFR (PDB: 1m17), and MAPK1 (PDB: 6ges)—were retrieved from the RCSB Protein Data Bank (https://www.rcsb.org/, accessed on 3 August 2025). The following positive control ligands were included: SD-36 (STAT3) [20], Alpelisib (PIK3CA) [21], SC79 (AKT1) [22], Gefitinib (EGFR) [23], and SCH772984 (MAPK1) [24]. Protein structures were prepared in PyMOL 3.1.3 by removing water molecules/ligands and adding hydrogens. Ligands were processed in AutoDock Tools 1.5.7 (Gasteiger charges, rotatable bonds, pdbqt conversion). A supervised docking strategy was employed, with the docking boxes constructed based on the positions of the co-crystal ligands. Docking was performed using AutoDock Vina 1.2.3. Binding energies were recorded, and interactions were visualized in 3D and 2D (Discovery Studio 2019). ADMET properties were predicted using SwissADME (http://www.swissadme.ch/, accessed on 5 August 2025), and toxicity profiles were assessed via ProTox-II (https://tox.charite.de/, accessed on 5 August 2025).

### 2.11. Molecular Dynamics Simulations (MDS)

Classical molecular dynamics (MD) simulations were performed using GROMACS 2023.5 to assess the stability of protein–ligand interactions identified through docking [25]. Protein PDB files were processed to generate topology structures, while ligand topologies were prepared using ACPYPE and integrated into the protein topology. The AMBER99SB-ILDN force field and TIP3P water model were applied. The system was placed in a cubic simulation box (1.0 nm), solvated with water, and neutralized with Na^+^/Cl^−^ ions. Energy minimization was performed with the steepest descent method at 300 K, followed by gradual heating under positional restraints. NVT and NPT equilibrations were conducted for 50,000 steps (2 fs/step) before the 100 ns production run. Temperature and pressure were maintained at 300 K (V-rescale thermostat) and 1 bar (Parrinello–Rahman barostat). Trajectories were recorded every 10 ps for analysis. The MM-PBSA method, using gmx_MMPBSA.py, was employed to estimate the binding free energy between the ligand and protein, assessing the energetics and interactions of the complexes [26].

### 2.12. Statistical Analysis

Data are expressed as mean ± SD (triplicate measurements). Group differences were analyzed using one-way ANOVA, followed by Duncan’s multiple range test (GraphPad Prism 8.0) to ensure robust statistical processing. Graphical representations were produced in GraphPad Prism 8.0 to enable precise, publication-quality figure design. Significance levels were set at *p* < 0.05 (significant) and *p* < 0.01 (highly significant).

## 3. Results

### 3.1. Antioxidant Activity and Enzyme Inhibition of ML, SG, and MLSG

**Figure 2:**
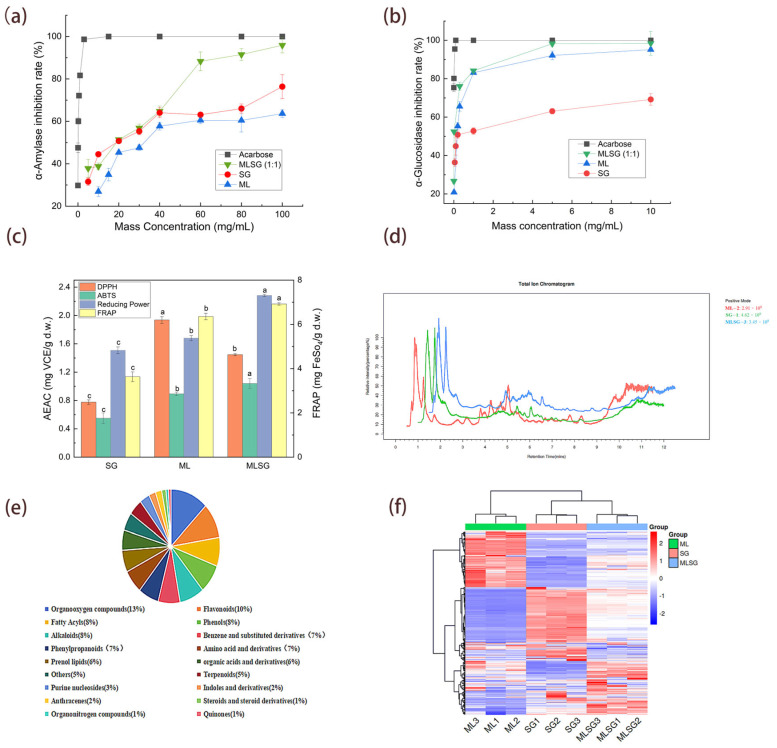
Enzyme inhibition, antioxidant capacity, and metabolic profiling of ML, SG, and MLSG. (**a**,**b**) Inhibition of α-amylase (**a**) and α-glucosidase (**b**) using ML, SG, MLSG, and acarbose (positive control) across a concentration range (mg/mL). (**c**) Antioxidant activities assessed using DPPH, ABTS^+^, reducing power, and FRAP assays, expressed as vitamin C equivalents (VCE, mg/g dry weight) or FeSO_4_ equivalents (mg/g dry weight). Different letters indicate significant differences (*p* < 0.05). (**d**) Representative total ion chromatograms (TICs) for ML, SG, and MLSG in positive ion modes. (**e**) Proportional distribution of identified metabolites among chemical categories. (**f**) Hierarchical clustering heatmap of differential metabolites (log_2_ fold change > 1, *p* < 0.05); red indicates higher and blue lower relative abundance.

The antioxidant capacity and enzyme inhibitory activities of mulberry leaf (ML), *Siraitia grosvenorii* (SG), and their combination (MLSG) were systematically evaluated (Figure 2). MLSG demonstrated potent dual-enzyme inhibition: α-Amylase: IC_50_ = 14.06 ± 0.55 mg/mL (CI = 0.58), 15.5-fold stronger than SG (18.34 ± 0.86 mg/mL) and 2.5-fold stronger than ML (34.75 ± 1.43 mg/mL) (Figure 2a, Table 1); α-Glucosidase: IC_50_ = 0.02 ± 0.001 mg/mL (CI = 0.12), 15.5-fold lower than SG (0.31 ± 0.12 mg/mL) and 5.5-fold lower than ML (0.11 ± 0.1 mg/mL). Notably, while MLSG’s α-glucosidase inhibition was 133-fold weaker than acarbose (0.000146 ± 0.000085 mg/mL), it represents the strongest efficacy among the natural extracts tested (Figure 2b, Table 1). This synergy (CI < 1) confirms MLSG’s potential as a botanical alternative to synthetic drugs. For antioxidant activity, MLSG displayed enhanced ABTS·+ scavenging (1.04 ± 0.07 mg VCE/g d.w.) and FRAP (6.92 ± 0.06 mg FeSO4/g d.w.) compared to individual extracts (ML: 0.89 ± 0.03, 6.35 ± 0.14; SG: 0.55 ± 0.07, 3.63 ± 0.22). While ML exhibited the highest DPPH activity (1.94 ± 0.05 mg VCE/g d.w.), MLSG still surpassed SG (0.78 ± 0.03 mg VCE/g d.w.) with intermediate potency (1.45 ± 0.02 mg VCE/g d.w.). Notably, MLSG demonstrated the strongest reducing power (2.28 ± 0.02 mg VCE/g d.w.), significantly exceeding both ML (1.68 ± 0.04) and SG (1.51 ± 0.05) (Figure 2c, Table 1).

### 3.2. Substance Analysis of ML, SG, and MLSG

To elucidate the enhanced efficacy of the MLSG formulation compared to individual ML or SG extracts, a UHPLC-Q-TOF-MS-based untargeted metabolomics approach was employed to characterize their chemical profiles. Total ion chromatograms (TICs) in positive/negative ion modes showed excellent signal stability in triplicate analyses, with overlapping metabolite TIC curves confirming consistent retention times and peak intensities (Figure 2d, Supplementary Figure S1). Metabolite identification was performed by matching accurate molecular weights, retention times, and MS/MS spectra against public databases, yielding 1290 annotated metabolites Supplementary Table S1), spanning 18 classes. Major classes included oxygenated organics (13%), flavonoids (10%), phenolic compounds (8%), alkaloids (8%), and fatty acyls (8%) (Figure 2e). Hierarchical clustering heatmap analysis revealed distinct accumulation patterns among ML, SG, and MLSG metabolites, highlighting significant differences among them (Figure 2f).

### 3.3. Multivariate Statistical Analysis

**Figure 3:**
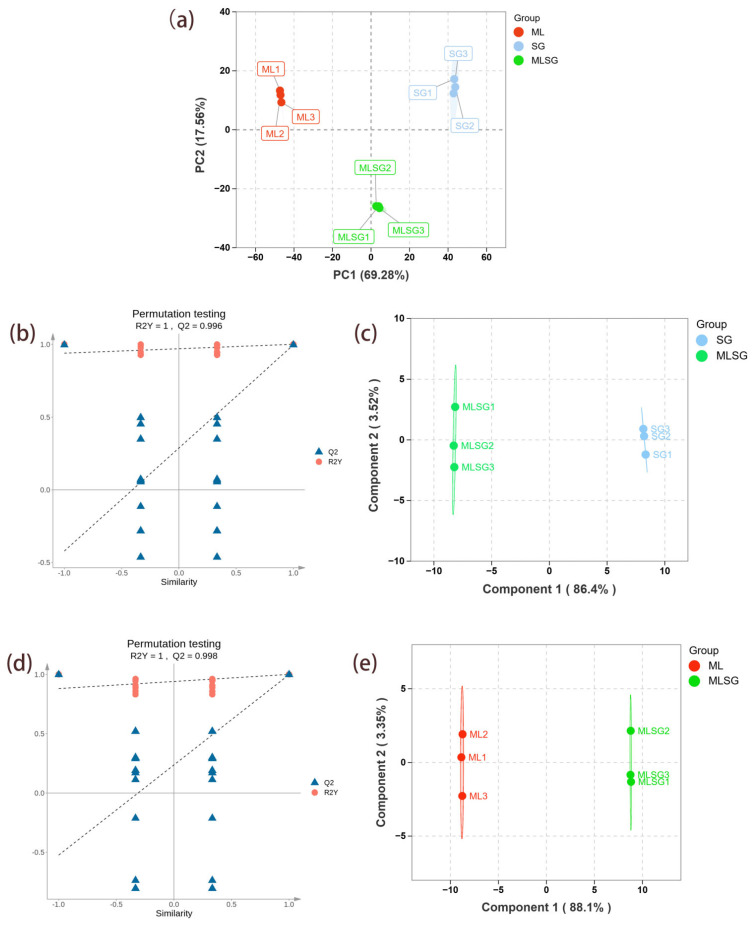
Multivariate statistical analysis of LC–MS metabolomic. (**a**) PCA score plot showing separation among *Morus alba* (ML), *Siraitia grosvenorii* (SG), and their combination (MLSG). (**b**) Permutation test validating the OPLS-DA model for MLSG vs. ML. (**c**) OPLS-DA score plot for MLSG vs. ML. (**d**) Permutation test validating the OPLS-DA model for MLSG vs. SG. (**e**) OPLS-DA score plot for MLSG vs. SG.

Principal component analysis (PCA) of the metabolomes of ML, SG, and MLSG revealed clear separation along the first principal component (PC1, explaining 69.28% of the total variance) and the second principal component (PC2, accounting for 17.57%). This suggests significant metabolic differences between the groups. OPLS-DA analysis (Figure 3b–e) further supported these distinctions. Specifically, the MLSG group exhibited clear differentiation from both the ML and SG groups, with the OPLS-DA model demonstrating strong goodness of fit and predictive performance: R^2^Y = 1, Q^2^ = 0.996 for MLSG vs. SG and R^2^Y = 1, Q^2^ = 0.998 for MLSG vs. ML. The permutation test results show that the original R^2^ and Q^2^ values were higher than those from the permuted models, supporting the robustness and validity of the models. These results suggest that MLSG presents a distinct metabolic profile compared to the individual components, providing statistical evidence for the observed differences.

### 3.4. Screening of Differentially Abundant Metabolites

**Figure 4:**
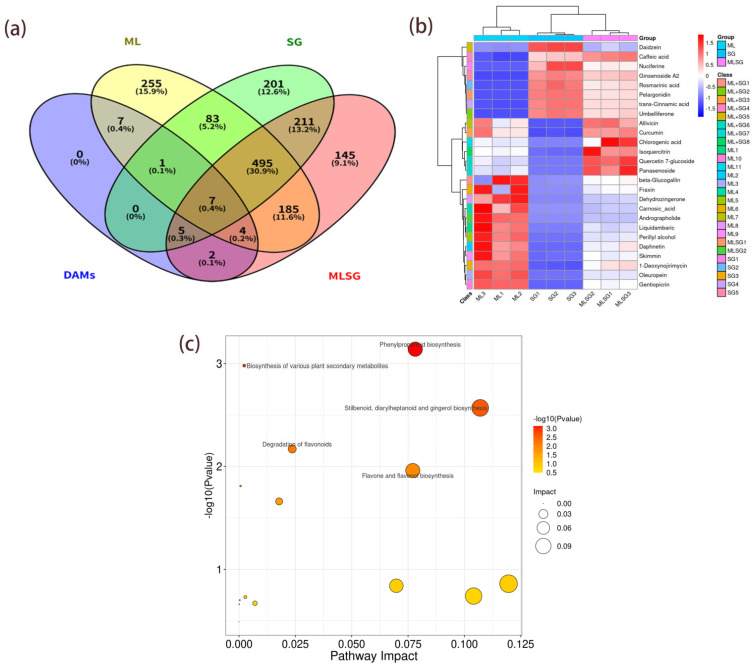
Comparative analysis of anti-diabetic differentially abundant metabolites (DAMs) from ML, SG, and MLSG. (**a**) Venn diagram showing the distribution of 26 bioactive DAMs with reported anti-hyperglycemic potential, classified into ML-specific, SG-specific, shared, and MLSG-unique metabolites. (**b**) Hierarchical clustering heatmap of the 26 DAMs, illustrating distinct metabolic profiles between MLSG and the individual extracts. (**c**) KEGG pathway enrichment of the 26 DAMs, highlighting predominant involvement in phenylpropanoid biosynthesis, flavonoid/flavone biosynthesis, and terpenoid pathways.

Differentially abundant metabolites (DAMs) were screened using thresholds of VIP > 1.25, *p* < 0.05, ppm ≤ 5 and |log_2_FC| > 1, identifying 144 DAMs in MLSG versus SG and 97 DAMs in MLSG versus ML (Tables S2 and S3). Among these, 26 metabolites exhibited potential anti-hyperglycemic activity based on the established literature (Table 2). Venn analysis classified these bioactive compounds into four groups: 11 ML-specific, 5 SG-specific, 8 shared constituents, and 2 MLSG-unique metabolites (Figure 4a). Hierarchical clustering further demonstrated distinct segregation of MLSG from individual extracts, confirming its unique metabolic profile (Figure 4b). KEGG enrichment revealed predominant involvement of these metabolites in phenylpropanoid biosynthesis, flavonoid/flavone biosynthesis, and terpenoid pathways (Figure 4c), collectively facilitating production of key hypoglycemic agents, including quercetin derivatives, alkaloids, and mogroside-type terpenoids.

### 3.5. Target Prediction of MLSG Against T2DM

**Figure 5:**
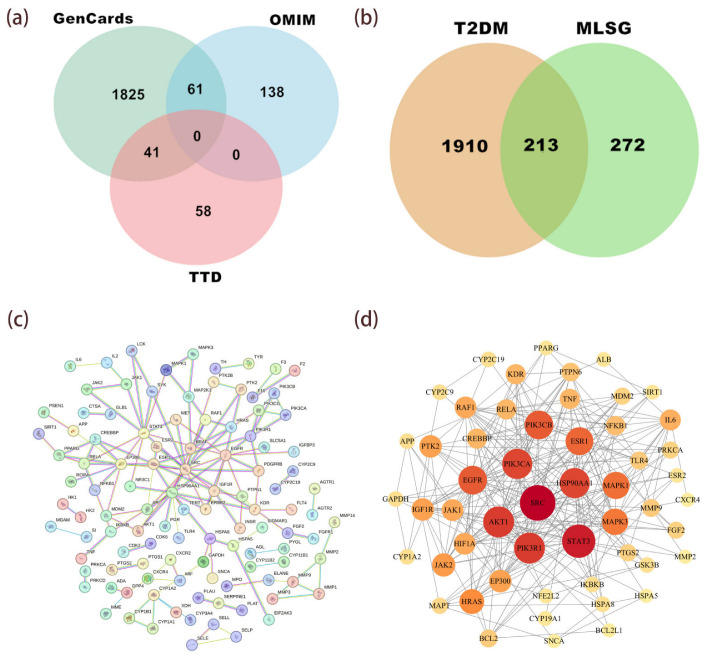
Network pharmacology analysis of MLSG against T2DM. (**a**) Venn diagram showing T2DM-related targets obtained from GeneCards, TTD, and OMIM databases. (**b**) Overlap between predicted MLSG targets and T2DM-associated targets, identifying 213 common targets. (**c**) Protein–protein interaction (PPI) network of the 213 intersecting targets generated via STRING database (confidence > 0.9). (**d**) Topological analysis highlighting the top anti-T2DM targets ranked by degree value, with node size proportional to degree and color intensity indicating higher connectivity.

Network pharmacology, an integrative approach combining systems biology and polypharmacology, elucidates drug–target interactions and predicts therapeutic mechanisms [27]. A total of 485 targets for 26 key compounds in the MLSG sample were obtained from the SwissTargetPrediction and SEA databases to identify targets for MLSG treatment of T2DM. In addition, 2123 T2DM-related targets were screened from the GeneCards, TTD, and OMIM databases (Figure 5a). Their intersection identified 213 potential targets related to the MLSG anti-diabetic agents (Figure 5b).

### 3.6. Protein–Protein Interaction (PPI) Network Analysis

For PPI protein interaction analysis (Figure 5c,d), we imported information on 213 drug–disease target intersections into the STRING database (reliability greater than 0.9). We used Cytoscape 3.10.0 software for visualization and the CytoNCA plugin for topological analysis. As the number of interacting targets increases, the color intensity of the targets varies. The network consists of 53 nodes and 372 edges, where each node represents a single protein target and each edge represents an interacting protein (Figure 5d). The size of the nodes is proportional to the degree value, and the saturation of the color map increases accordingly. The top 10 anti-T2DM targets ranked by degree value are SRC, STAT3, PIK3R1, AKT1, PIK3CA, HSP90AA1, PIK3CB, EGFR, ESR1, and MAPK1. Specific details of the most important targets are shown in Table 3.

### 3.7. GO and KEGG Enrichment Analysis of Potential T2DM Targets in MLSG

**Figure 6:**
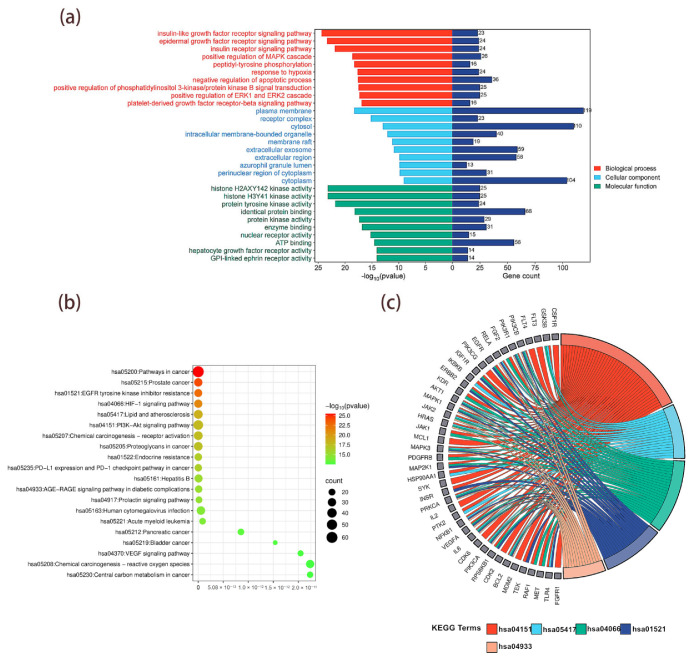
Functional enrichment analysis of common MLSG–T2DM targets. (**a**) GO enrichment analysis of 213 common targets shared between T2DM and MLSG, categorized into biological processes (red), cellular components (blue), and molecular functions (green), with the top 10 terms from each category ranked by significance (*p* < 0.05). (**b**) KEGG pathway enrichment bubble plot showing the top 20 significantly enriched pathways ranked by *p*-value, with bubble size representing the number of enriched genes and color scale indicating significance. (**c**) KEGG critical pathway–target network highlighting the top five pathways most closely associated with T2DM pathogenesis: PI3K–Akt signaling (hsa04151), lipid and atherosclerosis (hsa05417), HIF-1 signaling (hsa04066), EGFR tyrosine kinase inhibitor resistance (hsa01521), and AGE–RAGE signaling in diabetic complications (hsa04933), mapped to their associated targets.

The 213 common targets shared between T2DM and MLSG were subjected to GO and KEGG enrichment analyses using the DAVID database (Tables S4–S6). A total of 838 biological processes (BP) were significantly enriched (*p* < 0.05), including the insulin-like growth factor receptor signaling pathway, epidermal growth factor receptor signaling pathway, insulin receptor signaling pathway, response to hypoxia, and positive regulation of MAPK cascade. Cellular component (CC) analysis yielded 99 terms, such as plasma membrane, receptor complex, cytosol, intracellular membrane-bounded organelle, and membrane raft. For molecular function (MF), 240 terms were enriched, including histone H2AXY142 kinase activity, histone H3Y41 kinase activity, protein tyrosine kinase activity, identical protein binding, and enzyme binding. The top 10 enriched terms from each category (BP, CC, and MF) were selected based on significance (*p* < 0.05) and visualized (Figure 6a). The KEGG enrichment analysis identified 177 pathways (Table S7), and the top 20 pathways selected based on *p*-values were visualized as a bubble plot (Figure 6b). The top five pathways closely related to the pathogenesis of diabetes were visualized using a string diagram based on the number of enriched genes in each pathway. These pathways include the PI3K-Akt signaling pathway (hsa04151), lipid and atherosclerosis (hsa05417), the HIF-1 signaling pathway (hsa04066), EGFR tyrosine kinase inhibitor resistance (hsa01521), and the AGE-RAGE signaling pathway in diabetic complications (hsa04933) (Figure 6c).

### 3.8. Analysis of the “Component–Core Target–Signaling Pathway” Network

**Figure 7:**
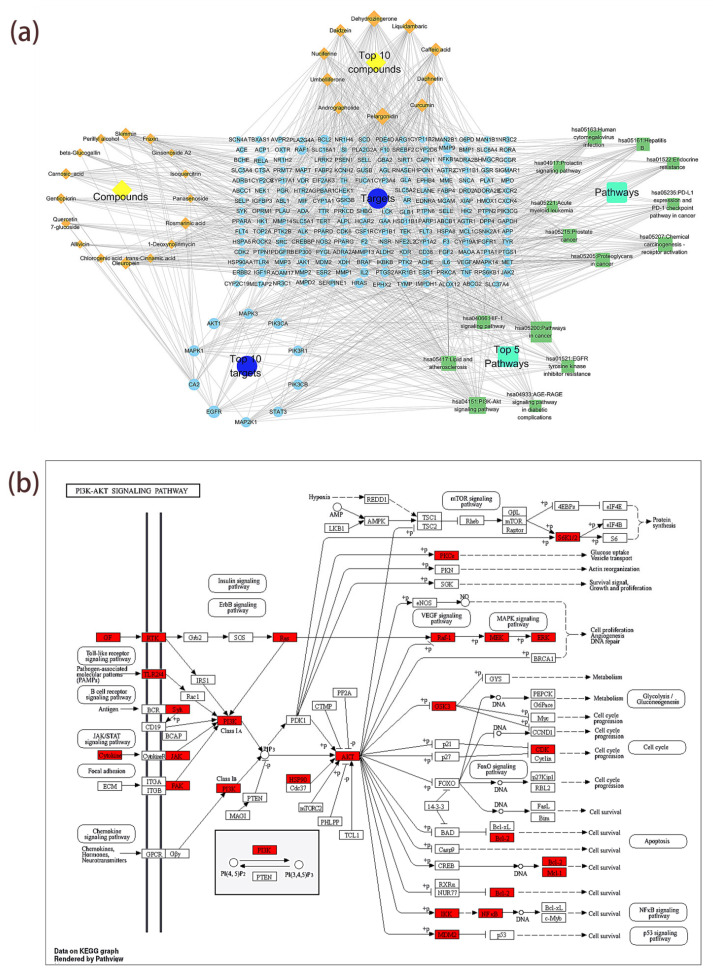
“Component–core target–signaling pathway” network analysis of MLSG against T2DM. (**a**) Network diagram linking 26 key bioactive compounds (orange diamonds) to 213 predicted protein targets (blue circles) and 15 KEGG-enriched pathways (green squares). Node size reflects degree value, with larger nodes indicating higher connectivity and stronger correlations; (**b**) KEGG PI3K–Akt signaling pathway map highlighting key MLSG-regulated targets (red), illustrating potential multi-target modulation relevant to T2DM pathogenesis.

To construct a compound–target–pathway network diagram and perform network analysis (Analyze Network), the targets of the top 15 KEGG enrichment pathways ranked by *p*-value and their corresponding compounds were imported into Cytoscape 3.10.0 software. The figure contains 26 compound nodes, 217 target nodes, 15 pathway nodes, and 1024 edges (Figure 7a). Larger node shapes indicate higher connectivity (degree), meaning the node has more connections with other nodes; all degree, betweenness, and closeness values for each node can be found in Table S8. Pelargonidin (degree = 47), dehydrozingerone (degree = 43), liquidambaric acid (degree = 36), and daphnetin (degree = 30) are the compounds with the highest connectivity. EGFR (degree = 24), CA2 (degree = 21), AKT1 (degree = 19), and MAPK1 (degree = 19) are the targets with the highest connectivity. The pathways with the highest connectivity are pathways in cancer (degree = 61), PI3K-Akt signaling pathway (degree = 43), lipids and atherosclerosis (degree = 35), and chemical carcinogenesis–receptor activation (degree = 34). Additionally, Figure 7b illustrates the key targets involved in the role of the PI3K-Akt signaling pathway in T2DM. This network shows that the compounds in MLSG can act on multiple targets and pathways, suggesting that these compounds may play an important role in alleviating type 2 diabetes by acting on these targets and pathways.

### 3.9. Analysis of Molecular Docking and ADMET Analysis

**Figure 8:**
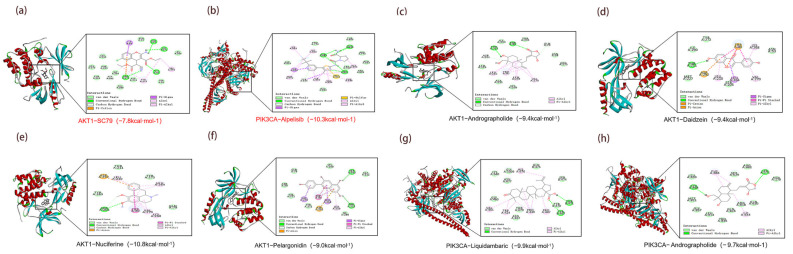
Molecular docking analysis of key MLSG metabolites with core protein targets. (**a**) AKT1-SC79 (positive control, −7.8 kcal/mol); (**b**) PIK3CA-alpelisib (positive control, −10.3 kcal/mol); (**c**) AKT1-andrographolide (−9.4 kcal/mol); (**d**) AKT1-daidzein (−9.4 kcal/mol); (**e**) AKT1-nuciferine (−10.8 kcal/mol); (**f**) AKT1-pelargonidin (−9.0 kcal/mol); (**g**) PIK3CA-liquidambaric acid (−9.9 kcal/mol); (**h**) PIK3CA-andrographolide (−9.7 kcal/mol).

Integrative analysis identified seven core targets (STAT3, PIK3R1, AKT1, PIK3CA, PIK3CB, EGFR, and MAPK1), with PIK3CA selected for docking due to its catalytic role. Docking of the top 10 MLSG metabolites against five core proteins showed strong affinities (–8 to –5 kcal/mol, Table 4), with AKT1-nuciferine (–10.8 kcal/mol) and PIK3CA-liquidambaric acid (–9.9 kcal/mol) outperforming known inhibitors (Figure 8e,g). Stable interactions, including hydrogen bonds, hydrophobic contacts, and aromatic stacking, were observed, suggesting that these metabolites may function as effective modulators of AKT1 and PIK3CA (Figure 8).

ADMET profiling of 26 metabolites indicated high gastrointestinal (GI) absorption for most, suggesting good bioavailability (Supplement Table S9). Pelargonidin, curcumin, and nuciferine showed strong absorption and low toxicity risks across hepatotoxicity, carcinogenicity, mutagenicity, and cytotoxicity. Daidzein and andrographolide exhibited moderate absorption but minimal toxicity, supporting their therapeutic potential. Additionally, curcumin and liquidambaric acid may interact with key CYP450 enzymes, influencing drug metabolism. These results align with the docking findings, highlighting metabolites like nuciferine and liquidambaric acid as promising candidates for further development due to their strong binding affinities and favorable pharmacokinetic properties.

### 3.10. Molecular Dynamics Simulations Results

Molecular dynamics simulations assessed the stability and binding affinity of key protein–ligand complexes, including the positive controls Alpelisib (PIK3CA) and SC79 (AKT1), alongside MLSG components. Six complexes were analyzed: (c) AKT1-andrographolide, (d) AKT1-daidzein, (e) AKT1-nuciferine, (f) AKT1-pelargonidin, (g) PIK3CA-liquidambaric acid, and (h) PIK3CA-andrographolide.

**Figure 9:**
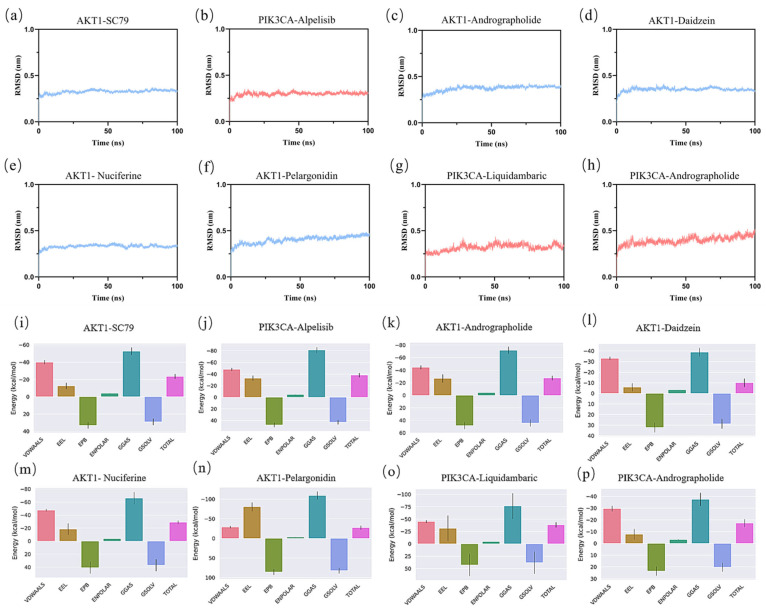
RMSD analysis of protein–ligand complexes over 100 ns simulation (**a**–**h**), and the total energy analysis of the protein–ligand complexes in MD (**i**–**p**). (**a**,**i**) AKT1-SC79, (**b**,**j**) PIK3CA-alpelisib, (**c**,**k**) AKT1-andrographolide, (**d**,**l**) AKT1-daidzein, (**e**,**m**) AKT1-nuciferine, (**f**,**n**) AKT1-pelargonidin, (**g**,**o**) PIK3CA-liquidambaric acid, and (**h**,**p**) PIK3CA-andrographolide complexes.

RMSD analysis (Figure 9a–h) showed stable binding across all complexes, with the fluctuation remaining below 0.1 nm during the last 30 ns, indicating that the structures of all eight complexes were stable. Notably, the PIK3CA-andrographolide and AKT1-nuciferine complexes exhibited the lowest RMSD values, suggesting the highest stability. RMSF analysis (Supplementary Figure S2) revealed that most amino acid residues in the complexes had RMSF values around 0.3 nm, indicating strong rigidity of the proteins and confirming their excellent stability. Rg (Supplementary Figure S3) and SASA (Supplementary Figure S4) analyses confirmed stable protein conformations with minimal fluctuations in solvent-accessible surface area.

Hydrogen bond analysis (Supplementary Figure S5) showed significant interactions, particularly in AKT1-SC79 and PIK3CA-alpelisib. Similarly, the MLSG complexes demonstrated strong hydrogen bonding, particularly in AKT1-nuciferine and PIK3CA-liquidambaric acid, indicating that these components also form stable interactions with key targets.

The Gibbs free energy values of the stable trajectories from the free energy calculations (Supplementary Figure S6) ranged from 0 to 17.5 kJ/mol, with each system exhibiting at least one minimum potential energy. These findings confirm that each complex system reached a stable state, making them suitable for further interaction analysis. DSSP analysis (Supplementary Figure S7) showed that the secondary structures of six protein—ligand complexes, along with the two positive controls, remained largely stable throughout the simulation, with no significant structural changes. The α-helix and β-sheet regions remained intact and continuous, with no evident unfolding or reconstruction, indicating good stability of the protein conformation under the simulation conditions.

MMPBSA calculations (Figure 9i–p and Table 5) revealed strong binding affinities for AKT1-nuciferine and PIK3CA-liquidambaric acid, with binding energies comparable to alpelisib (PIK3CA) and SC79 (AKT1). AKT1-andrographolide, AKT1-nuciferine, and AKT1-pelargonidin complexes exhibited stronger binding energies (−27.67, −28.99, and −27.27 kcal/mol) than the positive control AKT1-SC79 (−23.64 kcal/mol), indicating that these MLSG components bind more strongly to AKT1. Additionally, the PIK3CA-liquidambaric acid complex showed binding energy (−38.44 kcal/mol) nearly identical to that of PIK3CA-alpelisib (−38.20 kcal/mol), suggesting liquidambaric acid’s comparable or superior affinity for PIK3CA. These results highlight that MLSG components, including nuciferine, andrographolide, and liquidambaric acid, effectively modulate AKT1 and PIK3CA, key targets in insulin sensitivity and glucose metabolism. Overall, MLSG components demonstrate high binding affinity and stability, underscoring their potential as effective therapeutic agents for T2DM.

## 4. Discussion

Diabetes, a metabolic disease characterized by chronic high blood sugar levels, has emerged as a critical global health crisis, with type 2 diabetes (T2DM) being particularly burdensome because of the complex interplay of insulin resistance and beta cell dysfunction [28]. Mounting evidence confirms traditional Chinese medicine (TCM) offers distinct advantages in T2DM management via synergistic regulation of glucose–lipid metabolism [29]. Although the individual anti-glycation effects of mulberry leaves and *Siraitia grosvenorii* have been extensively studied, their combined anti-diabetic effects have not yet been revealed. This study showed that mulberry leaf and *Siraitia grosvenorii* mixed extract (MLSG) exhibited superior α-amylase and α-glucosidase inhibitory activity compared to individual extracts, with IC50 values of 14.06 mg/mL and 0.02 mg/mL, respectively. The combination index (<1) underscores the synergistic advantage. Similarly, MLSG exhibited enhanced overall antioxidant capacity compared with either extract alone, supporting the hypothesis that the combination may provide superior regulation of T2DM.

Untargeted UHPLC-QTOF-MS metabolomics identified 26 differentially expressed compounds with potential hypoglycemic activity across ML, SG, and MLSG (Table 2). These metabolites span diverse structural classes—terpenoids, flavonoids, phenylpropanoids, alkaloids, phenolic acids, and tannins—suggesting MLSG acts via multi-target, multi-pathway mechanisms. Among the flavonoids, quercetin glycosides (e.g., quercetin 7-glucoside, isoquercitrin, and panasenoside, derived from ML + SG) are widely recognized potent natural hypoglycemic agents [30,31]. Their mechanisms involve potent intestinal α-glucosidase inhibition [32] activation of the IRS-1/PI3K/Akt signaling pathway to enhance insulin sensitivity and GLUT4-mediated glucose uptake [33], and mitigation of insulin resistance through potent antioxidant and anti-inflammatory capacities [34]. Similarly, the isoflavone daidzein (ML + SG) protects β-cells and improves glucose homeostasis [35], while pelargonidin reduces hyperglycemia and oxidative stress by inhibiting hemoglobin glycation and free iron release [36]. Terpenoids constitute another significant hypoglycemic class. Oleuropein (ML-derived) enhances insulin sensitivity, protects β-cells, activates AMPK, and exerts strong antioxidant/anti-inflammatory effects [37]. Andrographolide targets diabetic nephropathy drivers (inflammation, oxidative stress, apoptosis) via STAT3/PI3K/Akt regulation [38]. Perillyl alcohol and Gentiopicrin (both ML-derived) also exhibit potential metabolic regulatory and antioxidant activities [39,40]. Among phenolic acids, chlorogenic acid (ML), caffeic acid (ML + SG), and rosmarinic acid (SG) inhibit α-glucosidase, improve insulin sensitivity, and protect islet cells [41,42]. Coumarin derivatives—daphnetin (ML) and umbelliferone (ML + SG)—ameliorate T2DM by inhibiting apoptosis and stimulating insulin secretion [43,44]. 1-Deoxynojirimycin (ML) functions like acarbose by delaying carbohydrate absorption [45], while nuciferine (SG) improves insulin resistance and modulates lipid metabolism [46]. Crucially, the MLSG combination (ML + SG/MLSG) revealed uniquely enriched or specific bioactive compounds, including curcumin, daidzein, multiple quercetin glycosides, pelargonidin, andrographolide, and beta-glucogallin. Curcumin is renowned for its multi-target hypoglycemic mechanisms involving potent anti-inflammatory/antioxidant effects, improved β-cell function, AMPK activation, and gluconeogenesis inhibition [47,48]. Beta-glucogallin likely contributes via antioxidant and potential enzyme inhibitory activities [49]. These combination-specific metabolites likely act through complementary or synergistic mechanisms (e.g., inhibiting carbohydrate absorption, enhancing insulin sensitivity, reducing inflammation/oxidative stress, protecting β-cells), forming the key chemical foundation for the superior hypoglycemic efficacy of the MLSG combination compared to individual ML or SG extracts.

Integrated network pharmacology and molecular docking elucidate that the 26 potential hypoglycemic compounds in MLSG may exert anti-type 2 diabetes mellitus (T2DM) effects by synergistically regulating core targets (STAT3, AKT1, PIK3CA, EGFR, and MAPK1) and modulating the AGE-RAGE, PI3K-Akt, and HIF-1 other signaling pathways. Mechanistically, (1) inhibition of the AGE-RAGE pathway can alleviate hyperglycemia-induced inflammatory damage and insulin resistance [50]; (2) functional restoration of the PI3K-Akt pathway, a hub for insulin signal transduction, directly ameliorates glucose metabolism disorders [51]; (3) modulation of the HIF-1 pathway may mitigate diabetes-associated hypoxic stress [52].

Molecular docking further confirmed high-affinity binding between key components and targets (binding energy ≤ −8 kcal/mol), with particularly strong interactions observed for AKT1-nuciferine (−10.8 kcal/mol), AKT1-daidzein (−9.5 kcal/mol), PIK3CA-andrographolide (−9.7 kcal/mol), and PIK3CA-liquidambaric acid (−9.9 kcal/mol). These complexes showed stable binding, supported by strong hydrogen bonds, hydrophobic interactions, and aromatic stacking (Figure 8), providing insight into the engagement of these compounds with their respective targets. When compared with classical ligands, the binding affinities of MLSG components were either comparable to or exceeded those of known inhibitors such as alpelisib (PIK3CA) and SC79 (AKT1), highlighting the potential of MLSG compounds to modulate these key pathways effectively. Additionally, ADMET profiling of these metabolites indicated high gastrointestinal (GI) absorption and favorable pharmacokinetic properties, supporting their potential for therapeutic use.

Molecular dynamics simulations of the selected protein–ligand complexes further validated the docking results (Figure 9, Table 5). The simulation trajectories confirmed the stability of the complexes over a 100 ns period, with the binding energies of key interactions remaining highly favorable. RMSD, RMSF, and other analyses indicated minimal structural deviations and flexibility, corroborating the stability and robust binding affinity of MLSG components. The results also suggest that these compounds can stabilize critical targets like AKT1 and PIK3CA, essential for regulating insulin sensitivity and glucose metabolism. Taken together, these findings underscore the potential of MLSG as a multi-target therapeutic agent for T2DM, combining high binding affinity, stability, and synergy in modulating metabolic pathways.

This study is the first to comprehensively evaluate the synergistic anti-T2DM effects of combining mulberry leaf and *Siraitia grosvenorii*, integrating enzymatic inhibition assays, antioxidant evaluation, metabolomics, network pharmacology, and comparative molecular docking with classical ligands. The main limitation is reliance on in vitro assays and in silico predictions without transcriptomic, cellular, or in vivo validation. Future work will incorporate multi-omics, cell-based functional assays, and animal studies to confirm efficacy, elucidate mechanisms, and assess safety.

## 5. Conclusions

This study reveals the enhanced efficacy of combining mulberry leaf and *Siraitia grosvenorii* (MLSG) in comparison to the individual extracts, particularly in enzyme inhibition and antioxidant activity. Through untargeted metabolomics, 26 potential hypoglycemic bioactive compounds were identified in the MLSG combination. Network pharmacology and molecular docking analyses further demonstrate that key components of MLSG (such as nuciferine, andrographolide, and liquidambaric acid) exert multi-target regulatory effects on T2DM by interacting with critical targets, such as AKT1 and PIK3CA, and modulating key signaling pathways, such as PI3K-Akt and AGE-RAGE. These findings offer novel insights into the therapeutic potential of MLSG for blood sugar management, laying a solid foundation for future research. However, further in vitro and in vivo studies are essential to validate the bioactive compounds, key targets, and mechanisms identified in this study.

## Figures and Tables

**Table 1:** Enzyme inhibition and antioxidant capacity of ML, SG, and MLSG.

	ML	SG	MLSG	Acarbose
α-AmylaseIC50 (mg/mL)	34.75 ± 1.43 a	18.34 ± 0.86 b	14.06 ± 0.55 c	0.066 ± 0.088 d
CI α-Amylase	/	/	0.58 < 1	/
α-GlucosidaseIC50 (mg/mL)	0.11 ± 0.1 b	0.31 ± 0.12 a	0.02 ± 0.001 c	0.000146 ± 0.00085 d
CI α-Glucosidase	/	/	0.12 < 1	/
DPPH (mg VCE/g d.w.)	1.94 ± 0.05 a	0.78 ± 0.03 c	1.45 ± 0.02 b	/
ABTS (mg VCE/g d.w.)	0.89 ± 0.03 b	0.55 ± 0.07 c	1.04 ± 0.07 a	/
RP (mg VCE/g d.w.)	1.68 ± 0.04 b	1.51 ± 0.05 c	2.28 ± 0.02 a	/
FRAP (mg FeSO_4_/g d.w.)	6.35 ± 0.14 b	3.63 ± 0.22 c	6.92 ± 0.06 a	/

CI: Combination index (synergy if < 1). Values expressed as mean ± SD. Different letters indicate a significant difference at the *p* < 0.05 level.

**Table 2:** List of 26 compounds that have potential anti-diabetic effects.

ID	Sample	Compounds	Class	mz	rt/s	ppm	Formula	Pos/Neg	Precursor
1	ML	Liquidambaric acid	Terpenoids	477.33	336.3	1.263	C_30_H_46_O_3_	pos	[M+Na]^+^
2	ML	Daphnetin	Phenylpropanoids	179.03	206.5	0.089	C_9_H_6_O_4_	pos	[M+H]^+^
3	ML	Oleuropein	Terpenoids	521.16	339.6	3.951	C_25_H_32_O_13_	neg	[M-H_2_O-H]^−^
4	ML	Carnosic_acid	Others	315.25	343.9	2.132	C_20_H_28_O_4_	pos	[M-H_2_O+H]^+^
5	ML	Perillyl alcohol	Prenol lipids	153.12	237.9	1.208	C_10_H_16_O	pos	[M+H]+
6	ML	1-Deoxynojirimycin	Alkaloids	144.06	54.8	1.996	C_6_H_13_NO_4_	neg	[M-H_2_O-H]^−^
7	ML	Allivicin	Flavonoids	611.15	244.7	4.998	C_27_H_30_O_16_	pos	[M+H]^+^
8	ML	Dehydrozingerone	Cinnamic acids and derivatives	175.14	293.5	0.105	C_11_H_12_O_3_	pos	[M-H_2_O+H]^+^
9	ML	Skimmin	Phenylpropanoids	325.09	218.1	0.954	C_15_H_16_O_8_	pos	[M+H]^+^
10	ML	Gentiopicrin	Terpenoids	417.10	221.2	0.109	C_16_H_20_O_9_	neg	[M+HCO_3_]^−^
11	ML	Chlorogenic acid	Organooxygen compounds	117.02	42.3	1.121	C_4_H_6_O_4_	neg	[M-H]^−^
12	SG	Nuciferine	Alkaloids	296.15	234.6	4.596	C_19_H_21_NO_2_	pos	[M+H]^+^
13	SG	Rosmarinic acid	Cinnamic acids and derivatives	383.18	151.6	2.047	C_18_H_16_O_8_	pos	[M+Na]^+^
14	SG	Fraxin	Phenylpropanoids	369.08	219.2	1.67	C_16_H_18_O_10_	neg	[M-H]^−^
15	SG	trans-Cinnamic acid	Phenylpropanoids	166.08	348.3	1.519	C_9_H_8_O_2_	pos	[M+NH_4_]^+^
16	SG	Ginsenoside A2	Prenol lipids	801.50	332.4	1.175	C_42_H_72_O_14_	pos	[M+H]^+^
17	ML+SG	beta-Glucogallin	Tannins	331.06	74	1.713	C_13_H_16_O_10_	neg	[M-H]^−^
18	ML+SG	Umbelliferone	Coumarins and derivatives	180.06	234.4	4.343	C_9_H_6_O_3_	pos	[M+NH_4_]^+^
19	ML+SG	Curcumin	Diarylheptanoids	369.12	273	1.906	C_21_H_20_O_6_	pos	[M+H]^+^
20	ML+SG	Caffeic acid	Cinnamic acids and derivatives	163.04	392.5	1.032	C_9_H_8_O_4_	pos	[M-H_2_O+H]^+^
21	ML+SG	Daidzein	Isoflavonoids	272.09	358.7	0.115	C_15_H_10_O_4_	pos	[M+NH_4_]^+^
22	ML+SG	Quercetin 7-glucoside	Flavonoids	465.10	288	1.179	C_21_H_20_O_12_	pos	[M+H]^+^
23	ML+SG	Panasenoside	Flavonoids	609.14	272.7	2.465	C_27_H_30_O_16_	neg	[M-H]^−^
24	ML+SG	Isoquercitrin	Flavonoids	463.08	280.9	4.309	C_21_H_20_O_12_	neg	[M-H]^−^
25	MLSG	Pelargonidin	Flavonoids	255.06	327.9	1.639	[C_15_H_11_O_5_]^+^	pos	[M-OH+H]^+^
26	MLSG	Andrographolide	Terpenoids	351.25	416.1	2.64	C_20_H_30_O_5_	pos	[M+H]^+^

**Table 3:** Topology parameters of important compounds and targets in the network.

	No.	Name	Degree	Betweenness	Closeness
Targets	1	SRC	34	2026.20	0.1469
Targets	2	STAT3	31	3294.25	0.1514
Targets	3	PIK3R1	28	821.50	0.1430
Targets	4	AKT1	28	3279.53	0.1512
Targets	5	PIK3CA	27	404.13	0.1418
Targets	6	HSP90AA1	27	2980.88	0.1497
Targets	7	PIK3CB	25	271.29	0.1400
Targets	8	EGFR	25	2270.09	0.1488
Targets	9	ESR1	24	2139.25	0.1496
Targets	10	MAPK1	23	1219.73	0.1476

**Table 4:** Binding energies of active components in MLSG and core targets (kcal/mol).

	STAT3 (PDB:6njs)	PIK3CA (PDB:4jps)	AKT1 (PDB:3o96)	EGFR (PDB:1m17)	MAPK1 (PDB:6ges)
Pelargonidin	−7.3	−6.6	−9.0	−7.4	−7.4
Dehydrozingerone	−5.7	−6.9	−5.1	−6.0	−5.0
Liquidambaric acid	−7.7	−9.9	−7.5	−8.9	−8.8
Daphnetin	−6.0	−6.8	−6.3	−6.6	−6.0
Curcumin	−7.5	−8.4	−7.2	−7.4	−8.7
Caffeic acid	−6.1	−7.0	−6.6	−5.5	−5.0
Umbelliferone	−5.9	−7.1	−6.4	−6.0	−6.0
Daidzein	−7.2	−8.0	−9.4	−7.2	−7.8
Nuciferine	−6.8	−8.4	−10.8	−8.7	−8.7
Andrographolide	−6.9	−9.7	−9.4	−7.9	−8.5
Classical ligands	−7.4	−10.3	−7.8	−8.0	−8.8

**Table 5:** Results of energy component delta (complex-receptor-ligand).

Energy Component (Kcal/mol)	AKT1- SC79	PIK3CA-Alpelisib	AKT1-Andrographolide	AKT1-Daidzein	AKT1-Nuciferine	AKT1-Pelargonidin	PIK3CA-Liquidambaric Acid	PIK3CA-Andrographolide
ΔVDWAALS	−40.05	−48.22	−44.79	−32.94	−47.55	−28.90	−45.03	−29.62
ΔEEL	−12.63	−32.93	−27.02	−5.93	−18.40	−80.38	−31.38	−7.85
ΔEPB	33.16	47.74	48.37	32.23	40.78	85.42	42.61	23.51
ΔENPOLAR	−4.12	−4.79	−4.22	−3.46	−3.83	−3.40	−4.64	−3.30
ΔEDISPER	0.00	0.00	0.00	0.00	0.00	0.00	0.00	0.00
ΔGGAS	−52.68	−81.15	−71.81	−38.87	−65.95	−109.29	−76.41	−37.46
ΔGSOLV	29.04	42.95	44.14	28.77	36.96	82.02	37.97	20.21
ΔTOTAL	−23.64	−38.20	−27.67	−10.10	−28.99	−27.27	−38.44	−17.26

## Data Availability

The data are contained within this article and its Supplementary Materials.

## Supplementary Materials

The following supporting information can be downloaded at https://www.mdpi.com/article/10.3390/antiox14091065/s1: Tables S1–S9 and Supplementary Information. Figure S1. TICs of Morus alba (ML), Siraitia grosvenorii (SG), and MLSG in negative-ion mode via UHPLC–QTOF–MS; Table S1. List of 1290 metabolites annotated in ML, SG, and MLSG extracts (positive/negative ion modes); Table S2. DAMs between MLSG vs. SG under VIP > 1.25, *p* < 0.05, mass error ≤ 5 ppm, |log_2_FC| > 1 (n = 144); Table S3. DAMs between MLSG vs. ML under the same criteria (n = 97); Table S4. GO Biological Process (BP) enrichment of 213 common targets (T2DM ∩ MLSG); Table S5. GO Cellular Component (CC) enrichment of 213 common targets; Table S6. GO Molecular Function (MF) enrichment of 213 common targets; Table S7. KEGG pathway enrichment of 213 common targets; Table S8. Network topology metrics for compound–target–pathway network: degree, betweenness centrality, and closeness centrality; Table S9. ADMET profiling of 26 candidate metabolites, including GI absorption, BBB permeability, CYP450 inhibition, hepatotoxicity, and carcinogenicity; Figure S2. RMSF of protein–ligand complexes over 100 ns MD simulations; Figure S3. Radius of gyration (Rg) of protein–ligand complexes over 100 ns MD simulations; Figure S4. SASA of protein–ligand complexes over 100 ns MD simulations; Figure S5. Hydrogen bond analysis for protein–ligand complexes during 100 ns MD simulations; Figure S6. Gibbs free-energy landscapes (FEL) of protein–ligand complexes; Figure S7. DSSP secondary structure evolution of proteins in complexes over 100 ns MD simulations.

## Abbreviations

The following abbreviations are used in this manuscript:

ML	*Morus alba* L. (Mulberry leaf)
SG	*Siraitia grosvenorii (Monk fruit)*
T2DM	Type 2 diabetes mellitus
MLSG	Mulberry leaf and *Siraitia grosvenorii* combination
STAT3	Signal transducer and activator of transcription 3
AKT1	RAC-alpha serine/threonine-protein kinase
PIK3CA	Phosphatidylinositol-4,5-bisphosphate 3-kinase catalytic subunit alpha
EGFR	Epidermal growth factor receptor
MAPK1	Mitogen-activated protein kinase 1
DNJ	1-Deoxynojirimycin
HFD	High-fat diet
CI	Combination index
PPI	Protein–protein interaction
ADMET	Absorption, distribution, metabolism, excretion, toxicity
MDS	Molecular dynamics simulations
DAMs	Differentially abundant metabolites
BP	Biological processes
CC	Cellular component
MF	Molecular function
