# Supplementary material for: Elucidating Key Components and Mechanisms Underlying the Synergistic Anti-Type 2 Diabetes Effect of Morus alba L. and Siraitia grosvenorii Combination: An Integrated In Vitro Enzymology, Untargeted Metabolomics, and Network Pharmacology Approach
Source: Antioxidants (Basel). 2025 Aug 29;14(9):1065. doi: 10.3390/antiox14091065 (PMC12466828; doi:10.3390/antiox14091065)
Supplement: Supplementary file 1 [file antioxidants-14-01065-s001.zip › Supplementary Information.pdf]

Supplementary Information

1.Here’s a concise summary of the software used in this research:

- **Cytoscape 3.10.0**
- **CytoNCA Plugin**: Use CytoNCA to calculate topological parameters and analyze key targets (Degree, Betweenness and Closeness).
- **PyMOL1.3:** Prepare protein structures, add hydrogens, remove water molecules.
- **AutoDock Tools 1.5.7:** Process ligands, set grid size (15 × 15 × 15 Å, spacing 1.0 Å), align grids with protein active sites.
- **AutoDock Vina 1.2.3:** Perform docking and record binding energies.
- **Discovery Studio 2019:** Visualize docking results in 3D and 2D.
- **GROMACS 2023.5:** Run simulations based on docking results, prepare topology files for proteins and ligands. The details are as follows：1.Simulation Time: 100 ns (production run); 2.Force Field: AMBER99SB-ILDN; 3.Water Model: TIP3P; 4.Simulation Box: 1.0 nm cubic box; 5.Ionization: Na+/Cl- ions for system neutralization; 6.Energy Minimization: Steepest descent method at 300 K; 7.Electrostatics Calculation: Particle Mesh Ewald (PME); 8.Temperature Control: 300 K (V-rescale thermostat); 9.Pressure Control: 1 bar (Parrinello-Rahman barostat); 10.Equilibration: NVT and NPT equilibrations for 50,000 steps (2 fs/step); 11.Trajectory Recording: Every 10 ps.
- **MMPBSA uses gmx_MMPBSA:**

$$\Delta G_{binding} = \Delta G_{complex} - \left(\Delta G_{protein} + \Delta G_{ligand}\right)$$

The following energy parameters are displayed in the MMPBSA calculation process:
ΔG_complex: Represents the total free energy of the protein-ligand complex. The free energy of the protein and ligand in solvent are represented by ΔG_protein and ΔG_ligand, respectively.
ΔVDWAALS: Interaction involving van der Waals forces.
ΔEEL: Electrostatic interaction.
ΔEPB: The polar contribution to solvation energy calculated using the PB method.
ΔENPOLAR: Contribution to solvation energy due to solute-solvent interactions in the PB model.
ΔEDISPER: Contribution to solvation energy due to attractive solute-solvent interactions in the PB model.
ΔG_gas: Molecular mechanics energy in the gas phase, considering all bonded and non-bonded interactions.
ΔG_solv: Total solvation energy calculated by the PB method.
ΔG: The total energy associated with binding. These values are expressed in kcal/mol.

- **GraphPad Prism 8.0**: Create graphs and statistical representations, set significance levels ($p < 0.05$ and $p < 0.01$).

2.The following is a graphic supplement to the manuscript:

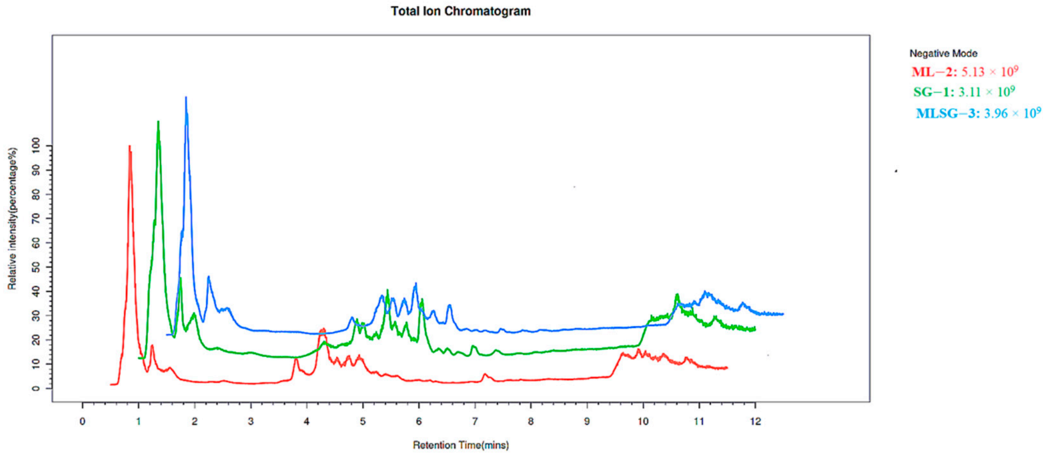

Figure S1. Representative total ion chromatograms (TICs) for ML, SG, and MLSG in negative ion modes.

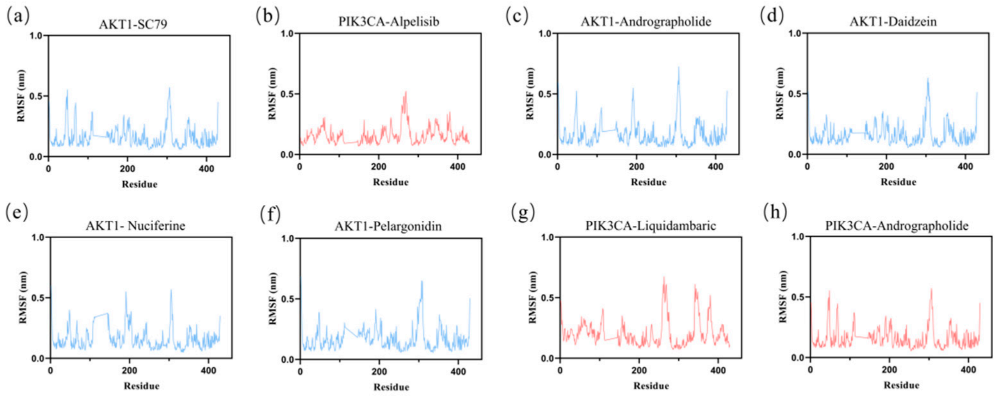

Figure S2. RMSF analysis of Protein-Ligand Complexes in MD Simulations. (a) AKT1-SC79, (b) PIK3CA-Alpelisib, (c) AKT1-Andrographolide, (d) AKT1-Daidzein, (e) AKT1-Nuciferine, (f) AKT1-Pelargonidin, (g) PIK3CA-Liquidambaric, and (h) PIK3CA-Andrographolide.

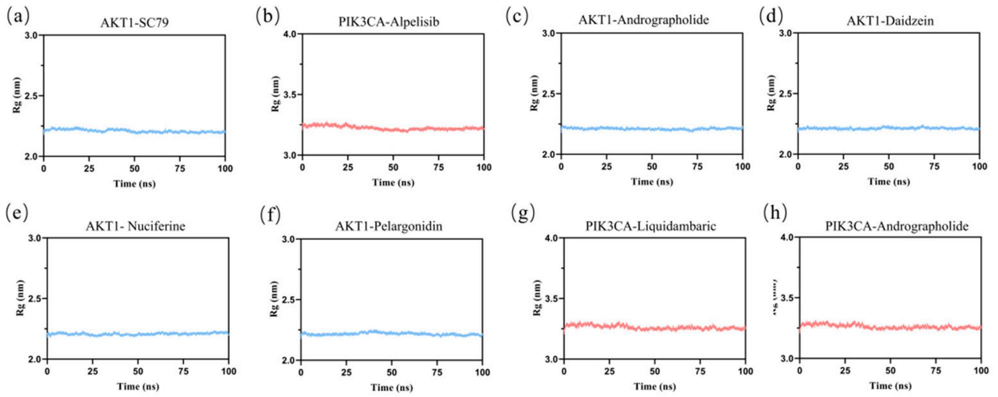

Figure S3. Radius of gyration (Rg) of the protein-ligand complexes over 100 ns simulation. (a) AKT1-SC79, (b) PIK3CA-Alpelisib, (c) AKT1-Andrographolide, (d) AKT1-Daidzein, (e) AKT1-Nuciferine,(f)AKT1-Pelargonidin,(g)PIK3CA-Liquidambaric,and(h)PIK3CA-Andrograph

olide.

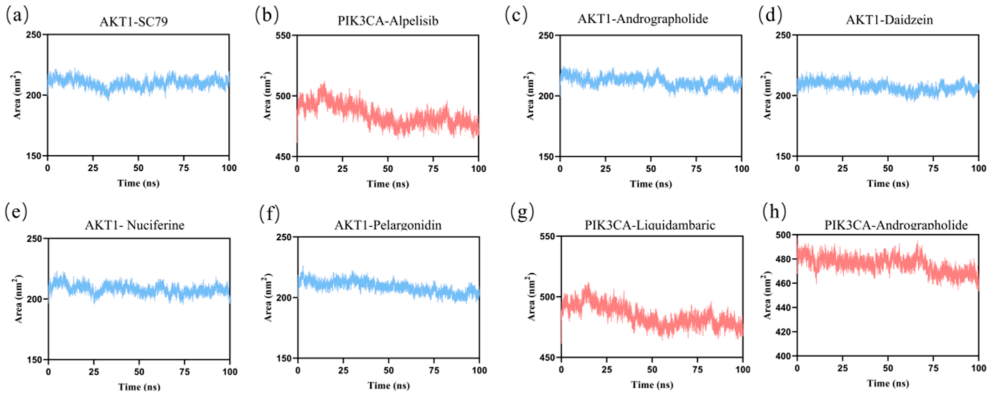

Figure S4. SASA of the protein-ligand complexes over 100 ns simulation. (a) AKT1-SC79, (b) PIK3CA-Alpelisib,(c) AKT1-Andrographolide, (d) AKT1-Daidzein, (e) AKT1-Nuciferine, (f) AKT1-Pelargonidin, (g) PIK3CA-Liquidambaric, and (h) PIK3CA-Andrographolide.

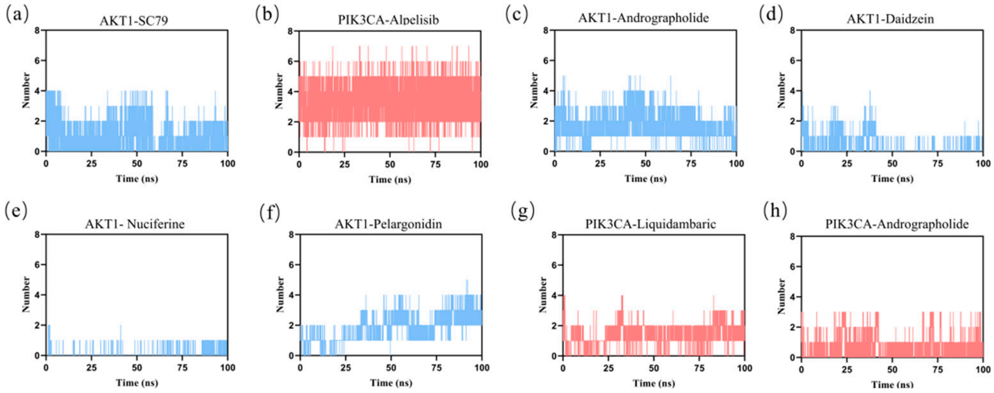

Figure S5. Hydrogen bond analysis of the Protein-Ligand Complexes. (a) AKT1-SC79, (b) PIK3CA-Alpelisib, (c) AKT1-Andrographolide, (d) AKT1-Daidzein, (e) AKT1-Nuciferine, (f) AKT1-Pelargonidin, (g) PIK3CA-Liquidambaric, and (h) PIK3CA-Andrographolide.

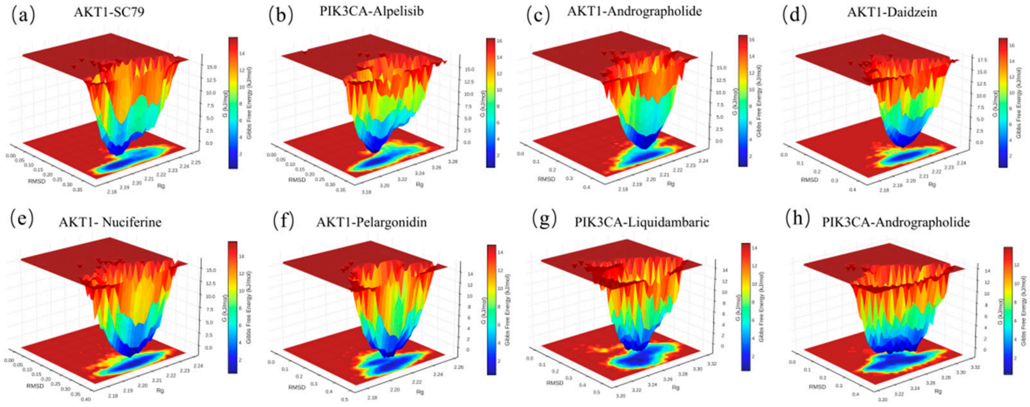

Figure S6. Gibbs free energy landscape of the Protein-Ligand Complexes. (a) AKT1-SC79, (b) PIK3CA-Alpelisib, (c) AKT1-Andrographolide, (d) AKT1-Daidzein, (e) AKT1-Nuciferine, (f) AKT1-Pelargonidin, (g) PIK3CA-Liquidambaric, and (h) PIK3CA-Andrographolide.

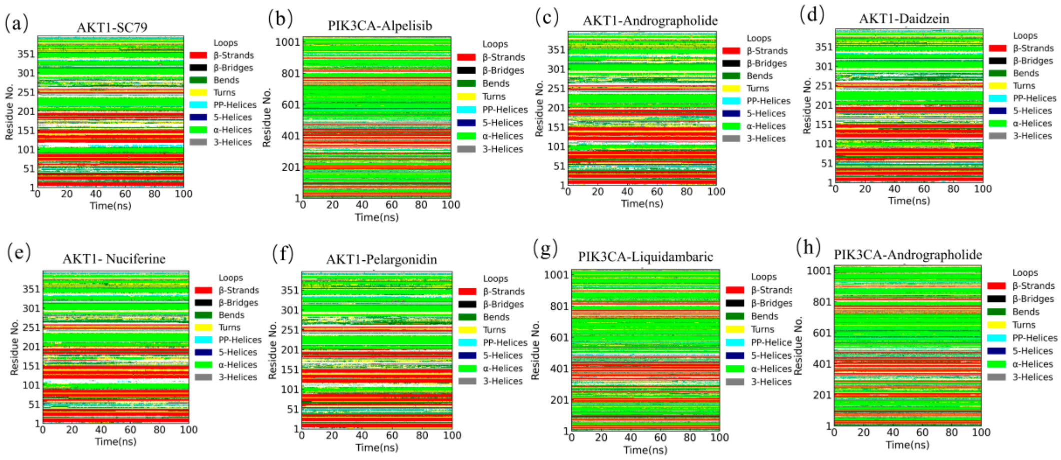

Figure S7. The DSSP of the Protein-Ligand Complexes in MD. (a) AKT1-SC79, (b) PIK3CA-Alpelisib, (c) AKT1-Andrographolide, (d) AKT1-Daidzein, (e) AKT1-Nuciferine, (f) AKT1-Pelargonidin, (g) PIK3CA-Liquidambaric, and (h) PIK3CA-Andrographolide.
